# Buccal Bone Thickness of Maxillary Incisors Adjacent to Palatally Impacted Canines: A Split-Mouth CBCT Study

**DOI:** 10.3390/medicina62061191

**Published:** 2026-06-20

**Authors:** Mehmet Gümüş Kanmaz, Genta Agani Sabah

**Affiliations:** 1 Department of Periodontology, Faculty of Dentistry, Izmir Tinaztepe University, Izmir 35400, Turkey; drmehmetkanmaz@gmail.com; 2 Department of Orthodontics, Faculty of Dentistry, Izmir Tinaztepe University, Izmir 35400, Turkey

**Keywords:** alveolar bone, buccal bone thickness, maxillary incisors, palatally impacted canine

## Abstract

*Background and Objectives*: To compare the buccal bone thickness of adjacent maxillary incisors between the impacted and contralateral control sides in patients with unilateral palatally impacted canines (PICs) using a split-mouth cone-beam computed tomography (CBCT) design. *Materials and Methods*: CBCT records of 26 patients with a unilateral PIC (18 females, 8 males; mean age, 17.35 ± 4.58 years) were retrospectively analyzed. Buccal bone thickness was measured at five equally spaced levels from the root apex (Level A) to the buccal alveolar crest (Level E) for the central and lateral incisors. Alveolar crest height, incisor torque and rotation, follicular width, canine localization, canine-to-root proximity, and root resorption were also assessed. *Results*: The impacted side showed significantly reduced buccal bone thickness at the two most apical levels of the lateral incisor: Level A (−0.81 mm; *p* < 0.001) and Level B (−0.35 mm; *p* = 0.004). No side differences were observed at the remaining lateral incisor levels or at any central incisor level. In the orientation-adjusted sensitivity model accounting for incisor torque and rotation, Level A remained significant (−0.75 mm; *p* < 0.001), whereas Level B was attenuated (*p* > 0.005). Lateral incisors on the impacted side also showed reduced labial torque (−4.97°; *p* = 0.001) and greater mesiobuccal rotation (−12.23°; *p* < 0.001). *Conclusions*: PICs were associated with localized apical reduction in buccal bone thickness of the adjacent lateral incisor, accompanied by reduced labial torque and greater mesiobuccal rotation. Buccal bone thickness may represent a relevant consideration during CBCT-based treatment planning for PICs.

## 1. Introduction

Maxillary canines are essential for facial esthetics, arch continuity, and functional occlusion [1]. Because of their long and complex eruption path, they are particularly susceptible to eruption disturbances and are regarded as the most commonly impacted teeth after third molars [2]. The reported incidence of maxillary canine impaction generally ranges from approximately 0.9% to 3%, and the condition has been described as more frequent in females [3]. Palatally impacted canines (PICs) constitute the majority of maxillary canine impactions, and have been linked to both guidance-related and genetic mechanisms [4].

PICs may remain undetected for a prolonged period and can therefore be associated with substantial complications affecting adjacent teeth and supporting tissues. These sequelae include displacement of neighboring teeth, external root resorption, cystic changes, ankylosis, and damage to periodontal and alveolar supporting structures [5,6,7]. Accurate localization of the impacted canine and precise assessment of its relationship with adjacent structures are therefore essential for diagnosis and treatment planning. The emergence of cone-beam computed tomography (CBCT) has markedly improved the diagnostic evaluation of impacted canines by enabling three-dimensional (3D) assessment of tooth position, root proximity, and associated resorptive changes without the superimposition and distortion inherent to conventional radiography [8,9,10].

Recent CBCT-based studies have demonstrated that unilateral canine impaction may be associated with positional and morphologic alterations in adjacent teeth when compared with the contralateral side. Such investigations have shown that adjacent lateral incisors and premolars may exhibit altered 3D position, root morphology, or volume in the presence of impacted canines [11,12,13,14]. Nevertheless, most of the available literature has focused on canine localization, adjacent root resorption, follicular characteristics, or tooth morphology, whereas the buccal bone thickness of the adjacent incisors is relatively underinvestigated. This represents an important gap, as localized buccal bone alterations may influence periodontal risk, biomechanical considerations, and combined orthodontic–surgical treatment planning. Taken together, these considerations highlight the need for a comprehensive CBCT-based assessment of alveolar bone morphology in incisors adjacent to impacted canines. Therefore, the aim of the present split-mouth CBCT study was to compare the buccal bone thickness of adjacent maxillary incisors between the impacted side and the contralateral control side, defined as the non-impacted side, in patients with unilateral PICs.

## 2. Materials and Methods

This retrospective study was approved by the Ethics Committee of Izmir Tinaztepe University (protocol number: MAEK2026/32). A split-mouth design was utilized to evaluate the maxillary central and lateral incisors. The impacted side served as the study group, whereas the contralateral control side, defined as the non-impacted side, served as the internal comparator. A priori sample size calculation was conducted based on pilot data from 10 patients with unilateral PICs who met the eligibility criteria for the present study. The effect size was derived from the partial eta squared for the Side × Tooth × Vertical interaction (the primary effect of interest in the planned LMM), which was 0.084, corresponding to a Cohen’s f of 0.303. With a 5% significance level, and a target power of 80%, the analysis indicated a minimum requirement of 15 patients. Ultimately, the final study sample consisted of 26 patients.

Pretreatment CBCT records were retrospectively retrieved from the Department of Orthodontics at Izmir Tinaztepe University. To reduce selection bias, CBCT records were screened consecutively in reverse chronological order, between February 2026 and January 2023, and all records meeting the eligibility criteria during this interval were included. The inclusion criteria were as follows: the presence of a unilateral PIC located mesial to the distal surface of the lateral incisor, which neither crossed the midline nor was positioned superior to the root apex; pretreatment CBCT records obtained before any orthodontic or surgical intervention for the impacted canine; fully erupted maxillary central and lateral incisors with clearly identifiable roots and apices; and the availability of high-resolution, diagnostic-quality CBCT scans. The CBCT field of view was required to include the entire maxillary anterior region, including both incisors, the impacted canine, alveolar crest, and root apices, and image quality had to be sufficient to identify the buccal cortical plate and root surfaces at all measurement levels. Patients were excluded if they had bilateral or buccal canine impactions; previous orthodontic treatment and/or surgical exposure, extraction, or traction attempt involving the impacted canine; a history of trauma or surgery in the maxillary anterior region; missing permanent teeth except third molars; peg-shaped lateral incisors; craniofacial syndromes; cysts, tumors, periapical lesions, supernumerary teeth, odontomas, or other local pathological conditions in the maxillary anterior region; periodontal bone loss or inflammatory bone defects affecting the maxillary incisors; systemic diseases; other dental anomalies apart from the impacted canine; or CBCT scans with motion artifacts, metallic artifacts, incomplete field of view, or poor visualization of the buccal cortical plate. This selection process resulted in a final sample of 26 Caucasian patients with a unilateral PIC, comprising 18 females (mean age at the time of radiographic acquisition: 15.69 ± 3.57 years) and 8 males (mean age: 21.09 ± 4.57 years).

CBCT images were acquired using the MyRay Hyperion X9 Pro 3D system (Cefla, Imola, Italy) with exposure parameters set at 90 kV, 8 mA, and 26.4 s. All images were exported in DICOM format and analyzed using 3D Slicer software (version 5.10.0; https://www.slicer.org; accessed on 10 April 2026). Volumes were visualized using multiplanar reconstruction (MPR), with 0.11 mm isotropic voxel size. Linear measurements were recorded in millimeters (mm) and angular measurements in degrees (°). All radiographic assessments were performed by a single investigator (G.A.S.) using a standardized orientation and measurement protocol with predefined anatomical landmarks, measurement levels, and classification criteria, in order to reduce measurement bias and limit observer-related variability.

**Figure 1:**
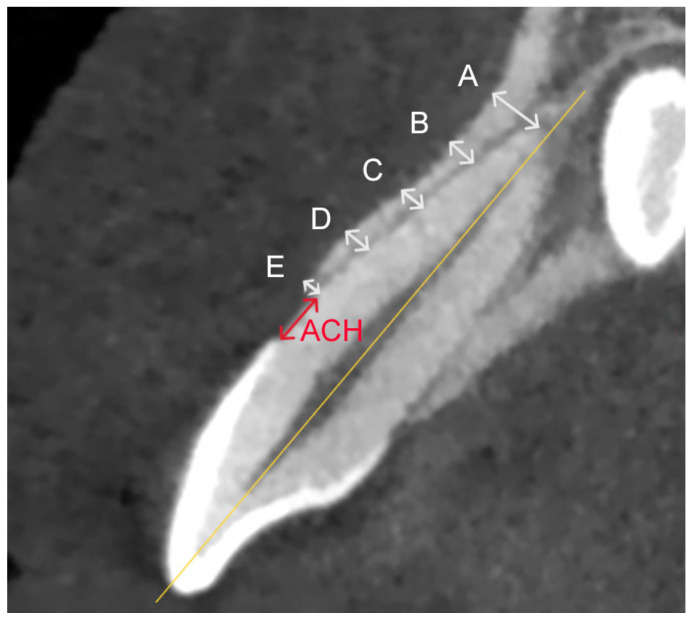
Sagittal CBCT slice illustrating the buccal bone thickness and ACH measurement methodology. Yellow line: the tooth’s longitudinal axis. White double-headed arrows: buccal bone thickness measurements at five levels, A (root apex) to E (buccal alveolar crest), and B, C, D at equal intervals between A and E. Red double-headed arrow: ACH measurement.

Buccal bone thickness was assessed on tooth-specific sagittal slices [15]. Using the Reformat module in 3D Slicer, each slice was carefully oriented to pass through the center of the respective incisor and its alignment with the true long axis was confirmed when the slice displayed the maximum visible root length. Afterwards five reference lines were constructed perpendicular to the long axis of the incisor (the line passing through the incisal edge to the root apex) to define buccal bone thickness measurement levels along the root. First, a crestal reference line (Line E) was positioned at the buccal alveolar crest (defined as the most coronal point of the buccal cortical plate adjacent to the tooth). Second, an apical reference line (Line A) was positioned at the root apex. The distance between Lines E and A was then divided into four equal segments by three intermediate parallel lines (Lines B, C, and D), thereby creating five levels from the apex to the crest (A, B, C, D and E). At each level, buccal bone thickness was measured as the linear distance from the outer buccal root surface to the outer surface of the buccal cortical plate. Additionally, vertical alveolar crest height (ACH) was measured as the linear distance from the cementoenamel junction (CEJ) to the buccal alveolar crest, parallel to the tooth’s long axis (Figure 1).

**Figure 2:**
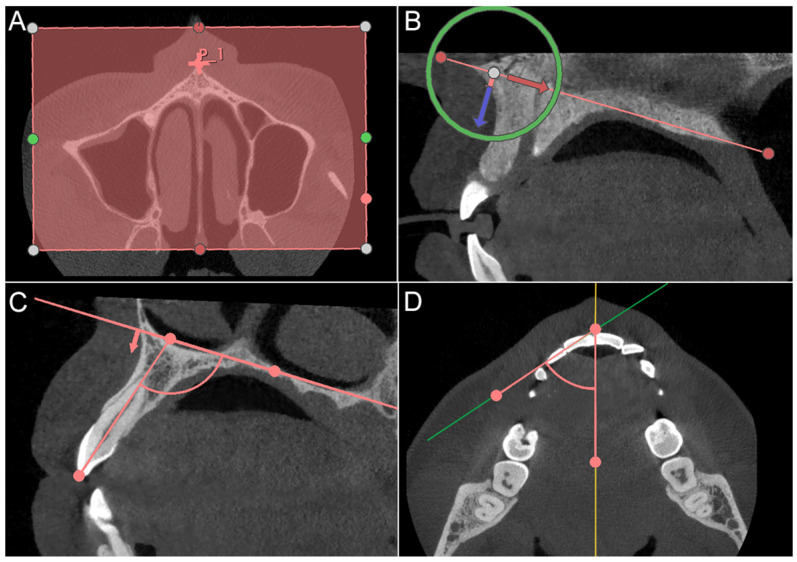
CBCT images illustrating torque and rotation measurements. (**A**) Axial view of the palatal reference plane (red overlay). (**B**) Sagittal view of the palatal reference plane (red line). (**C**) Sagittal view illustrating the measurement of maxillary incisor torque. (**D**) Axial view illustrating the measurement of maxillary incisor rotation, defined as the angle between a tangent line drawn along the incisal edge of the lateral incisor (green line) and the midpalatal reference line (yellow line).

A palatal reference plane was constructed in the Markups module of 3D Slicer using the three-point plane method. The plane was defined by placing three anatomical landmarks at the anterior nasal spine (ANS), posterior nasal spine (PNS), and an additional point on the hard palate located lateral to the ANS–PNS line. The resulting plane was saved and locked to prevent inadvertent movement during subsequent measurements. To evaluate maxillary incisor torque, the tooth long axis was established as a line connecting the incisal edge and root apex. Maxillary incisor torque was then quantified as the angle formed between this long axis and the palatal reference plane. Higher torque values indicated greater labial inclination of the incisor. Incisor rotation was assessed on axial CBCT slices as the angle between a tangent line drawn along the incisal edge of the incisor and the midpalatal reference line. Rotation was measured separately for the central and lateral incisors on both the impacted and control sides. Lower angular values indicated greater mesiobuccal rotation of the incisor [13] (Figure 2).

The width of the dental follicle associated with the impacted canine was evaluated in both sagittal and axial slices, where the maximum follicular width was measured as the greatest linear distance from the outer enamel surface of the canine crown to the inner cortical border surrounding the follicle. The presence and severity of root resorption on the adjacent incisors were also evaluated using the classification system described by Ericson and Kurol [5]. Resorption was graded on a four-point scale: Grade 0, no resorption (intact root surface, but the cementum layer may be lost); Grade 1, slight resorption (loss of the cementum layer, with the resorption reaching no further than half the dentin thickness); Grade 2, moderate resorption (loss of dentin exceeding half the thickness, but the pulp lining remains intact); and Grade 3, severe resorption (resorption reaching or exposing the pulp).

The spatial position of the impacted canine was classified according to its horizontal (mesiodistal) and vertical localization, and proximity to the adjacent incisors. Horizontal localization of the impacted canine crown tip was assessed on axial CBCT slices and categorized into two regions relative to the adjacent incisors: the lateral incisor region, when the crown tip was located within the mesiodistal boundaries of the lateral incisor, and the central incisor region, when the crown tip was located within the mesiodistal boundaries of the central incisor. The vertical position of the impacted canine crown tip was evaluated on sagittal CBCT slices relative to the long axis of the adjacent incisor. The root of the adjacent incisor was divided into three equal segments, allowing the canine’s vertical level to be classified as being in a cervical, middle, or apical position. To assess the relationship between the impacted canine and the roots of the adjacent incisors, their proximity was examined across multiplanar CBCT slices and classified into three categories: no contact, characterized by the presence of visible alveolar bone separating the impacted canine from the adjacent incisor root; follicle contact, defined as contact limited to the dental follicle of the impacted canine against the adjacent root; and direct contact, defined as physical contact between the crown of the impacted canine and the root of the adjacent incisor.

To assess intra-examiner reliability, 30% of the total sample (*n* = 8) was randomly selected and re-measured by the same investigator (G.A.S.) after a two-week interval.

### Statistical Analysis

Descriptive statistics were summarized as means and standard deviations (SD) or medians and interquartile ranges (IQR) for continuous variables, and as frequencies and percentages for categorical variables. Intra-examiner reliability for continuous measurements was evaluated using intraclass correlation coefficients (ICCs) based on a two-way mixed-effects model with absolute agreement, whereas weighted kappa (κ) was used for ordinal categorical data.

Patient age, sex, and tooth orientation, including incisor torque and rotation, were considered as potential factors that could influence buccal bone thickness. Because the study used a split-mouth design, comparisons between the impacted and control sides were made within the same patient, thereby reducing confounding from patient-level characteristics such as age and sex. Nevertheless, to additionally adjust for the association between age and alveolar bone dimensions across the age range of the sample, age was included as a fixed-effect covariate in the primary LMM. An additional sensitivity analysis was performed by including sex as a fixed-effect covariate. Sex was not significantly associated with buccal bone thickness and did not materially change the primary findings. Therefore, it was not retained in the final model for reasons of model parsimony. Tooth orientation was addressed separately in an orientation-adjusted sensitivity LMM by adding incisor torque and rotation as covariates.

Buccal bone thickness was analyzed using the primary LMM. Side, tooth type, vertical increment, and their two- and three-way interactions were entered as fixed effects, with age included as a covariate and patient ID as a random intercept. Pairwise comparisons of estimated marginal means (EMMs) were performed to compare the impacted and control sides within each tooth type and vertical increment, with a Bonferroni-corrected significance threshold of *p* ≤ 0.005. The secondary orientation-adjusted sensitivity LMM was considered exploratory and was used only to assess whether the primary buccal bone thickness findings were robust after accounting for incisor orientation.

ACH, maxillary incisor torque and rotation were each analyzed using separate LMMs with side, tooth type, their interaction, and age as fixed effects, and patient ID as a random intercept. Pairwise comparisons of EMMs were used to compare the impacted and control sides separately for the central and lateral incisors.

To explore whether follicular dimensions were associated with the buccal bone thickness alterations at the lateral incisor, Spearman rank-order correlation coefficients (ρ) were calculated between follicle width and lateral incisor buccal bone thickness at Levels A and B, where significant differences were identified in the primary analysis.

All statistical analyses were performed using IBM SPSS Statistics (version 26; IBM Corp., Armonk, NY, USA) with significance set at *p* < 0.05 unless otherwise specified.

## 3. Results

Descriptive statistics regarding demographic variables, follicle width, root resorption, spatial position of the impacted canine, and proximity to adjacent incisors are summarized in Table 1. Root resorption was observed predominantly at the lateral incisor on the impacted side, where 38.5% of teeth showed some degree of resorption. In contrast, only 11.5% of central incisors showed slight resorption. Canine-to-root proximity was unevenly distributed, particularly for the lateral incisor, where most cases showed direct contact and only one case showed no contact.

Regarding intrarater reliability, ICC indicated excellent agreement for both the central and lateral incisors for buccal bone thickness (ranging from 0.981 to 0.994 across all vertical increments), ACH (ranging from 0.932 to 0.943), torque (ranging from 0.954 to 0.968), rotation (ranging from 0.947 to 0.953) and good agreement for follicle width (ICC = 0.879). Weighted κ indicated almost perfect agreement across all measurements, including root resorption grading (κ = 0.998), canine-to-root contact classification (κ = 0.997), canine vertical position (κ = 0.981), and canine mesiodistal position (κ = 0.987).

**Figure 3:**
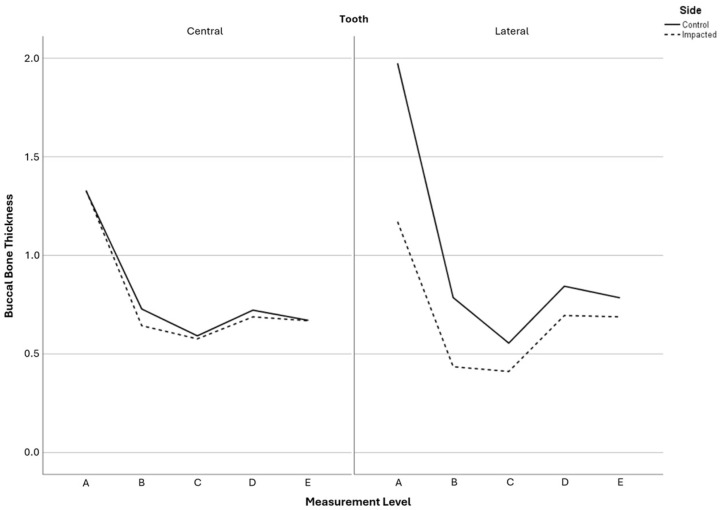
Mean buccal bone thickness (mm) at five vertical measurements levels (A: apex, E: crest, B, C, and D: equally spaced intermediate levels between the root apex and the buccal alveolar crest) for the central and lateral incisors on the control and impacted sides.

**Figure 4:**
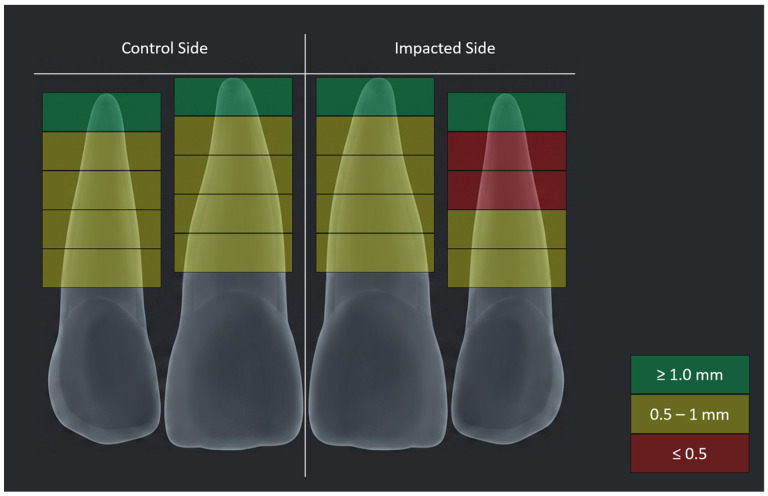
Schematic illustration of buccal bone thickness categories at five vertical measurement levels for the maxillary central and lateral incisors on control and impacted sides. Green indicates buccal bone thickness ≥ 1.0 mm, yellow indicates 0.5–1.0 mm, and red indicates ≤ 0.5 mm.

Preliminary sensitivity analysis indicated that sex was not significantly associated with buccal bone thickness (*p* > 0.05) and did not alter the main effects. Thus, it was excluded from the final primary model. Within this final primary LMM, there were significant main effects of side (*p* < 0.001) and vertical increment (*p* < 0.001), whereas the main effect of tooth type was not significant (*p* > 0.05). Age was not significantly associated with buccal bone thickness (*p* > 0.05). Significant two-way interactions were observed for side × tooth (*p* < 0.001), side × vertical increment (*p* = 0.028), and tooth × vertical increment (*p* = 0.046). The three-way interaction between side, tooth type, and vertical increment was also significant (*p* = 0.019), indicating that the side-related difference in buccal bone thickness varied according to both tooth type and vertical measurement level. Pairwise comparisons showed that buccal bone thickness was significantly reduced on the impacted side only at the two most apical levels of the lateral incisor. At Level A, the impacted side showed a mean reduction of 0.81 mm compared with the control side (*p* < 0.001), while at Level B the reduction was 0.35 mm (*p* = 0.004). No side differences were observed at Levels C, D, or E of the lateral incisor, where mean differences did not exceed 0.16 mm, or at any level of the central incisor, where mean differences did not exceed 0.09 mm (*p* > 0.005) (Table 2; Figure 3). Schematic illustration of the buccal bone thickness categories at five vertical measurement levels for the maxillary central and lateral incisors on the impacted and control sides is shown in Figure 4.

A secondary orientation-adjusted sensitivity LMM was performed by additionally including incisor torque and rotation as covariates. Because of the modest sample size, this model was considered exploratory and was used only to evaluate whether the primary buccal bone thickness findings were robust after accounting for incisor orientation. In this model, the three-way interaction remained significant (*p* = 0.016). Torque was significantly associated with buccal bone thickness (*p* < 0.001), whereas age and rotation were not (*p* > 0.05). Pairwise comparisons showed that the reduction at the lateral incisor Level A remained significant after adjustment for torque and rotation (mean difference = −0.746 mm; *p* < 0.001), whereas the Level B difference was attenuated (mean difference = −0.288 mm; *p* > 0.005) and did not meet the Bonferroni-corrected significance threshold.

The LMM for ACH revealed no significant effect of side and the side × tooth interaction (*p* > 0.05). A significant main effect of tooth type was observed (*p* < 0.001), reflecting a greater CEJ-to-crest distance at the lateral incisor compared to the central incisor regardless of side. Although a slight trend toward increased crestal distance (0.24 mm) was observed for lateral incisors on the impacted side, pairwise comparisons confirmed that the presence of the impacted canine did not significantly alter the vertical bone level for either the central or lateral incisors (*p* > 0.05). Conversely, the LMM for maxillary lateral incisor torque demonstrated a significant main effect for side (*p* = 0.001), with incisors on the impacted side exhibiting reduced labial torque (105.93°) compared to the control side (110.17°). Although the side × tooth interaction was not significant (*p* > 0.05), simple main effects analysis indicated that the torque alterations were more pronounced at the lateral incisor (mean difference = 4.97°; *p* = 0.001) than at the central incisor (mean difference = 2.34°; *p* > 0.05). Incisor rotation revealed significant main effects of side (*p* = 0.009) and tooth type (*p* < 0.001), as well as a significant side × tooth interaction (*p* = 0.008). Pairwise comparisons showed no side difference for the central incisor (mean difference: 0.15°; *p* = 0.963), whereas the lateral incisor demonstrated lower rotational angle on the impacted side (mean difference: −12.23°; *p* < 0.001), indicating greater mesiobuccal rotation (Table 3). Spearman correlation analysis showed no significant correlation between dental follicle width and buccal bone thickness at Level A (ρ = 0.247; *p* = 0.224) or Level B (ρ = 0.296; *p* = 0.142) of the lateral incisor on the impacted side.

## 4. Discussion

The present split-mouth CBCT study compared the buccal bone thickness of adjacent maxillary incisors between the impacted and control sides in patients with unilateral PICs. To the best of our knowledge, this is the first study to investigate this relationship. The results show that a PIC is associated with significant reduction of the buccal bone thickness overlying the adjacent lateral incisor relative to the contralateral non-impacted side. This difference was specifically localized to the apical measurement levels, with mean reductions of 0.81 mm at Level A and 0.35 mm at Level B. However, after additional adjustment for incisor torque and rotation, the Level A difference remained significant, whereas the Level B difference was attenuated. These findings expand the current understanding of the local effects of canine impaction, which has traditionally focused on root resorption and positional displacement of neighboring teeth [5,6,13], by introducing alveolar bone thickness as an additional clinically relevant consideration.

Although numerous CBCT-based studies have investigated the buccal bone dimensions of maxillary anterior teeth, these have primarily been conducted in the context of implant planning and in periodontally healthy populations. The systematic review and meta-analysis by Tsigarida et al. [16] synthesized data from 50 studies and reported that the majority of maxillary anterior teeth have a buccal bone thickness less than 1 mm, with lateral incisors consistently demonstrating the thinnest buccal bone among the anterior teeth. Our findings on the control side are broadly consistent with these normative values, with the mean buccal bone thickness of the lateral incisor measuring 0.79 mm at the crestal level (Level E) and ranging between 0.56 and 0.85 mm at the mid-root levels (C and D). On the impacted side, however, the apical buccal bone thickness was reduced to 1.16 mm compared with 1.97 mm on the control side, indicating that the apical region, which is physiologically the thickest part of the buccal plate, showed the greatest absolute side-to-side difference in the presence of a PIC.

Previous CBCT investigations have demonstrated that PICs are associated with altered alveolar bone dimensions on the impacted side [17,18,19]. However, these studies have predominantly assessed buccopalatal width at the canine site rather than the buccal bone thickness of the adjacent incisors. Tadinada et al. [17] reported reduced buccopalatal alveolar width at 2 mm from the alveolar crest on the impacted side, with no difference at the 6 mm or 10 mm levels, a finding subsequently corroborated by Prashanth and Durgekar [18]. Dadgar et al. [19] similarly observed reduced alveolar thickness at 2 mm in both buccally and palatally impacted groups, with no difference at deeper levels in palatal impactions and even greater thickness at 10 mm in buccal impactions. Collectively, these studies suggest that alveolar bone alterations associated with canine impaction may be depth-dependent rather than uniform. The present study extends these observations by demonstrating that the buccal bone thickness of the adjacent lateral incisor is also subject to site-specific thinning in the presence of a PIC. Notably, whereas the aforementioned studies identified the crestal region as the most affected level, the primary analysis in the present study localized the side-to-side difference to the apical levels of the lateral incisor, particularly Level A, which remained significant after additional adjustment for incisor torque and rotation. This difference likely reflects the fundamentally different anatomical sites evaluated, with previous studies measuring total buccopalatal width at the canine region, whereas the present study assessed buccal bone thickness along the adjacent incisors’ root.

The presence of a PIC was not associated with a significant difference in ACH for either the central or lateral incisors. Regardless of side, however, ACH was consistently greater at the lateral incisor than at the central incisor. The ACH observed for lateral incisors in the present study (1.53 mm on the control side and 1.77 mm on the impacted side) falls at the lower end of the normative ranges reported by Tsigarida et al. [16] (1.90–3.54 mm) for healthy populations. This may be related to the younger mean age of the current sample (17.35 years), consistent with the age-related increase in CEJ-to-crest distance reported previously [16].

The site-specific reduction in buccal bone thickness identified at the apical root levels of the lateral incisor on the impacted side may be related, at least in part, to the concurrent positional findings, as lateral incisors on the impacted side exhibited both significantly reduced labial torque and greater mesiobuccal rotation compared with the control side. These observations are consistent with previous CBCT findings. Dekel et al. [13] similarly reported both buccal root torque and mesiobuccal rotation in lateral incisors adjacent to PICs, while Light et al. [20] demonstrated that palatal impactions are associated with labial root displacement of the ipsilateral incisors, with the effect being confined to the impacted side. Therefore, a plausible interpretation is that the palatally positioned canine may be associated with buccal displacement and rotational alteration of the lateral incisor, which could contribute to compression of the overlying buccal bone and localized thinning at the apical levels. However, because the Level A difference remained significant after additional adjustment for incisor torque and rotation, incisor orientation alone is unlikely to fully account for the observed buccal bone reduction. The finding that the central incisor exhibited smaller and non-significant torque and rotation differences, accompanied by no buccal bone thickness alteration at any measurement level, further suggests that the magnitude of buccal bone reduction may be related to the degree of adjacent-tooth displacement.

The pattern of lateral incisor displacement and localized apical buccal bone reduction may also be interpreted in relation to the anatomical proximity between the impacted canine and the adjacent incisor root. In the present study, the majority of the lateral incisors on the impacted side demonstrated direct physical contact (76.9%) with the PIC, and 38.5% exhibited concurrent root resorption, consistent with earlier reports [5,6,21,22,23]. Furthermore, the vertical position of the impacted canine relative to the lateral incisor was situated at the middle or apical root level in 88.0% of cases, with cervical positioning observed in only 12.0%. This distribution corresponds to the anatomical region where buccal bone reduction was identified. Although the limited number of observations in some subcategories precluded formal statistical comparisons, the frequent co-occurrence of direct contact, root resorption, and apical bone thinning may support a pressure-driven anatomical mechanism. In this context, direct contact between the PIC and the adjacent lateral incisor root may contribute both to external root resorption and to buccal displacement of the lateral incisor root, thereby compressing the overlying buccal cortical plate. The absence of a significant association between follicle width and apical buccal bone thickness on the impacted side further suggests that follicular expansion alone is unlikely to fully explain the observed thinning. These findings are consistent with Dekel et al. [13] and Lam et al. [14], who reported that physical contact between the canine crown and adjacent roots may be an important factor in neighboring tooth displacement. Future studies with larger and more balanced subgroups are needed to clarify the relative contribution of these anatomical factors.

Taken together, the present findings carry several significant implications for the understanding and clinical management of PICs. While previous research has largely focused on canine localization, root resorption, and adjacent tooth displacement, the present findings extend this perspective by suggesting that the local alveolar bone environment of the adjacent lateral incisor may also be altered, particularly in the apical region, which is anatomically the thickest portion of the buccal cortical plate in the anterior maxilla. The integration of buccal bone thickness with alveolar crest height, incisor torque, and incisor rotation within a split-mouth analytical framework therefore contributes to a more comprehensive characterization of the dentoalveolar effects associated with PICs. This site-specific reduction is clinically relevant and should be considered during CBCT-based assessment of PICs, particularly when planning surgical exposure and orthodontic traction, as it may influence decisions regarding bracket placement, force-application vectors, and the timing of alignment. Furthermore, in cases where the treatment plan involves the extraction of the lateral incisor and subsequent implant placement, the compromised buccal bone thickness must be carefully accounted for during surgical planning. Chappuis et al. [24] identified the facial bone wall thickness as the most critical factor influencing post-extraction bone resorption and classified sites into two distinct phenotypes: thin-wall phenotypes (≤1 mm), which exhibited progressive bone resorption with a median vertical bone loss of 7.5 mm, and thick-wall phenotypes (>1 mm), which showed only minor resorption with a median vertical loss of 1.1 mm within 8 weeks of healing. Accordingly, bone augmentation procedures or socket preservation strategies may need to be integrated into the treatment plan for thin-wall phenotypes to counteract the expected dimensional tissue alterations and ensure adequate bone volume for predictable implant placement with favorable esthetic outcomes. Nonetheless, treatment planning for impacted canines is multifactorial, and buccal bone thickness should be interpreted as one component of the overall evaluation rather than as a stand-alone determinant.

Regarding the strengths of this study, the split-mouth design, in which the contralateral control side served as the internal comparator, reduced interindividual variability and enabled direct within-patient comparison of the incisors adjacent to unilateral PICs. This approach has been endorsed for orthodontic investigations by Pandis et al. [25] and has been used in analogous impaction studies by Albayrak and Senisik [26] and Leonardi et al. [11]. In addition, the study incorporated a five-level buccal bone thickness measurement protocol spanning the entire root length and included complementary assessment of ACH, incisor torque, and incisor rotation, together with descriptive evaluation of additional impaction-related CBCT findings.

Nonetheless, several limitations should be acknowledged. First, the retrospective cross-sectional design precludes causal inference, and the reduced buccal bone thickness observed at the apical level of the lateral incisor may be confounded by pre-existing anatomical variations other than torque and rotation. Second, although the split-mouth design reduced interindividual variability, the control side cannot be regarded as a perfectly equivalent biological control. Because buccal bone thickness levels were defined relative to each tooth’s long axis, side-to-side differences in tooth orientation could theoretically influence the anatomical location sampled. Although incisor torque and rotation were measured bilaterally and included in an orientation-adjusted sensitivity analysis to address this concern, residual effects of tooth-specific morphology or measurement-plane variation cannot be entirely excluded. Other potentially relevant factors, including periodontal phenotype, arch form, dental crowding, and pre-existing buccal bone morphology, could not be fully quantified due to the study design. Third, although eligible CBCT records were screened consecutively, the use of a CBCT-based sample from a single center may have introduced selection and referral bias, since patients undergoing CBCT imaging may not fully represent the broader population of patients with PICs. Fourth, although the final sample size exceeded the minimum requirement estimated by the a priori power analysis, the inclusion of only 26 patients remains a limitation. The modest sample size may limit the statistical power, particularly for subgroup analyses related to canine contact type, root resorption, vertical localization, and follicle width. Therefore, these findings should be interpreted cautiously, and future studies with larger samples and more balanced anatomical subgroups are needed to confirm the present results. Fifth, all measurements were performed by a single investigator, who could not be blinded because the impacted side was inherently identifiable on CBCT images. Although this may have enhanced internal consistency, it precluded assessment of inter-examiner reliability and may have introduced measurement and observer bias. Similarly, the categorical grading of canine-to-root contact and root resorption is subject to detection and classification bias. Sixth, many buccal bone thickness values were below 1 mm, a range in which CBCT measurement accuracy is reduced [27]. Therefore, small absolute differences should be interpreted cautiously. Finally, the study included only Caucasian subjects, and given the documented ethnic variations in alveolar bone morphology [16], the findings may not be directly generalizable to other populations. Future prospective studies with larger and more diverse samples, balanced anatomical subgroups, and standardized clinical and three-dimensional records are needed to confirm these findings.

## 5. Conclusions

In conclusion, this split-mouth CBCT study showed that unilateral PICs were associated with localized reduction in buccal bone thickness overlying the adjacent lateral incisor relative to the control side, with the greatest difference observed at the apical level. Lateral incisors on the impacted side also demonstrated reduced labial torque and greater mesiobuccal rotation. Although these positional differences may contribute to the observed buccal bone reduction, the persistence of the Level A difference after adjustment for torque and rotation suggests that incisor orientation alone does not fully account for the finding. Buccal bone assessment may therefore represent a relevant additional component of CBCT-based evaluation of PICs, together with the broader clinical and radiographic factors that guide treatment planning.

## Figures and Tables

**Table 1:** Sample characteristics and descriptive data of CBCT parameters.

Demographics (*n* = 26)	Gender	Female, *n* (%)	18 (69.2)
		Male, *n* (%)	8 (30.8)
	Age (years)	Mean ± SD	17.35 ± 4.58
		Median (IQR)	15.75 (7.72)
		Min–Max	12.30–26.75
Follicle width (*n* = 26)		Mean ± SD	3.33 ± 1.43
		Median (IQR)	3.40 (2.80)
		Min–Max	1.00–4.90
Root resorption	Central (*n* = 26), *n* (%)	Grade 0	23 (88.5)
		Grade 1	3 (11.5)
		Grade 2	0 (0.0)
		Grade 3	0 (0.0)
	Lateral (*n* = 26), *n* (%)	Grade 0	16 (61.5)
		Grade 1	5 (19.2)
		Grade 2	1 (3.8)
		Grade 3	4 (15.4)
Horizontal position (*n* = 26), *n* (%)		Central Incisor Region	18 (69.2)
		Lateral Incisor Region	8 (30.8)
Vertical position	Central (*n* = 17), *n* (%)	Cervical	4 (23.5)
		Middle	11 (64.7)
		Apical	2 (11.8)
	Lateral (*n* = 25), *n* (%)	Cervical	3 (12.0)
		Middle	14 (56.0)
		Apical	8 (32.0)
Proximity	Central (*n* = 26), *n* (%)	No contact	9 (34.6)
		Follicle contact	12 (46.2)
		Direct contact	5 (19.2)
	Lateral (*n* = 26), *n* (%)	No contact	1 (3.8)
		Follicle contact	5 (19.2)
		Direct contact	20 (76.9)

SD: Standard Deviation; IQR: interquartile range; Min: Minimum; Max: Maximum. Vertical position was assessed only for incisors showing follicle contact or direct contact with the impacted canine. Therefore, the denominators for central (*n* = 17) and lateral (*n* = 25) vertical-position classifications differ from the total sample size.

**Table 2:** Buccal bone thickness (mm) by side, tooth type, and vertical increment.

		Control Side (mm) EMM (SE)	Impacted Side (mm) EMM (SE)	Mean Difference (mm) 95% CI (Lower; Upper)	*p* Value
Central Incisor	A	1.33 (0.09)	1.32 (0.09)	−0.01 −0.249; 0.234	0.953
	B	0.73 (0.09)	0.64 (0.09)	−0.09 −0.333; 0.150	0.459
	C	0.59 (0.09)	0.57 (0.09)	−0.02 −0.262; 0.221	0.866
	D	0.73 (0.09)	0.68 (0.09)	−0.05 −0.287; 0.196	0.710
	E	0.68 (0.09)	0.66 (0.09)	−0.02 −0.257; 0.226	0.900
Lateral Incisor	A	1.97 (0.09)	1.16 (0.09)	−0.81 −1.052; −0.569	<0.001 *
	B	0.79 (0.09)	0.44 (0.09)	−0.35 −0.595; −0.112	0.004 *
	C	0.56 (0.09)	0.41 (0.09)	−0.15 −0.392; 0.091	0.222
	D	0.85 (0.09)	0.69 (0.09)	−0.16 −0.400; 0.083	0.198
	E	0.79 (0.09)	0.68 (0.09)	−0.11 −0.352; 0.131	0.370

EMM: estimated marginal mean; SE: standard error; CI: confidence interval. A: root apex; E: buccal alveolar crest; B, C, and D: equally spaced intermediate levels between the root apex and the buccal alveolar crest. Mean differences were calculated as impacted side minus control side; negative values indicate reduced buccal bone thickness on the impacted side. * Significant after Bonferroni correction at *p* ≤ 0.005.

**Table 3:** Comparison of ACH, incisor torque and rotation between incisors of the impacted and control sides.

		Control Side EMM (SE)	Impacted Side EMM (SE)	Mean Difference 95% CI (Lower; Upper)	*p* Value
ACH (mm)	Central Incisor	1.35 (0.09)	1.32 (0.09)	−0.03 −0.275; 0.211	0.754
	Lateral Incisor	1.53 (0.09)	1.77 (0.09)	0.24 −0.014; 0.468	0.069
Maxillary Incisor Torque (°)	Central Incisor	108.66 (1.42)	106.32 (1.42)	−2.34 −5.275; 0.585	0.113
	Lateral Incisor	111.23 (1.42)	106.26 (1.42)	−4.97 −7.898; −2.038	0.001 *
Maxillary Incisor Rotation (°)	Central Incisor	79.53 (2.94)	79.68 (2.94)	0.15 −6.224; 6.520	0.963
	Lateral Incisor	54.11 (2.94)	41.88 (2.94)	−12.23 −18.607; −5.863	<0.001 *

EMM: estimated marginal mean; SE: standard error; CI: confidence interval; ACH: alveolar crest height. Mean differences were calculated as impacted side minus control side. For ACH, a positive difference indicates a greater CEJ-to-crest distance on the impacted side. For torque, a negative difference indicates a reduced angle between the tooth long axis and palatal reference plane on the impacted side. For rotation, a negative difference indicates a lower rotational angle on the impacted side. * Statistically significant at *p* < 0.05.

## Data Availability

The data that support the findings of this study are available from the corresponding author upon reasonable request.

## Abbreviations

The following abbreviations are used in this manuscript:

3D	Three-dimensional
ANS	Anterior nasal spine
ACH	Alveolar Crest Height
CBCT	Cone-beam computed tomography
CEJ	Cementoenamel junction
CI	Confidence interval
DICOM	Digital Imaging and Communications in Medicine
ICC	Intraclass correlation coefficient
IQR	Interquartile range
LMM	Linear mixed model
Min	Minimum
Max	Maximum
MPR	Multiplanar reconstruction
PIC	Palatally impacted canine
PNS	Posterior nasal spine
SD	Standard deviation
